# Phage-Driven Loss of Virulence in a Fish Pathogenic Bacterium

**DOI:** 10.1371/journal.pone.0053157

**Published:** 2012-12-31

**Authors:** Elina Laanto, Jaana K. H. Bamford, Jouni Laakso, Lotta-Riina Sundberg

**Affiliations:** 1 Centre of Excellence in Biological Interactions, Universities of Jyväskylä and Helsinki, Finland; 2 Department of Biological and Environmental Science and Nanoscience Center, University of Jyväskylä, Jyväskylä, Finland; 3 Department of Biosciences, University of Helsinki, Helsinki, Finland; 4 Department of Biological and Environmental Science, University of Jyväskylä, Jyväskylä, Finland; University of York, United Kingdom

## Abstract

Parasites provide a selective pressure during the evolution of their hosts, and mediate a range of effects on ecological communities. Due to their short generation time, host-parasite interactions may also drive the virulence of opportunistic bacteria. This is especially relevant in systems where high densities of hosts and parasites on different trophic levels (e.g. vertebrate hosts, their bacterial pathogens, and virus parasitizing bacteria) co-exist. In farmed salmonid fingerlings, *Flavobacterium columnare* is an emerging pathogen, and phage that infect *F. columnare* have been isolated. However, the impact of these phage on their host bacterium is not well understood. To study this, four strains of *F. columnare* were exposed to three isolates of lytic phage and the development of phage resistance and changes in colony morphology were monitored. Using zebrafish (*Danio rerio*) as a model system, the ancestral rhizoid morphotypes were associated with a 25–100% mortality rate, whereas phage-resistant rough morphotypes that lost their virulence and gliding motility (which are key characteristics of the ancestral types), did not affect zebrafish survival. Both morphotypes maintained their colony morphologies over ten serial passages in liquid culture, except for the low-virulence strain, Os06, which changed morphology with each passage. To our knowledge, this is the first report of the effects of phage-host interactions in a commercially important fish pathogen where phage resistance directly correlates with a decline in bacterial virulence. These results suggest that phage can cause phenotypic changes in *F. columnare* outside the fish host, and antagonistic interactions between bacterial pathogens and their parasitic phage can favor low bacterial virulence under natural conditions. Furthermore, these results suggest that phage-based therapies can provide a disease management strategy for columnaris disease in aquaculture.

## Introduction

Over the last few years, there has been a growing concern regarding the emergence of disease outbreaks in livestock. It has become clear that environmental changes (e.g., climate warming, human intervention, enhanced transmission by transportation, use of antibiotics) have resulted in the development of new pathogens and diseases, and in addition, diseases that were previously under control have re-emerged [1]–[3]. In particular, intensive farming environments have been found to be evolutionary hot spots for pathogens. For example, the ecological and epidemiological features of intensive farming, including high host densities, effective transmission, and potential for serial passage, can select for high virulence of pathogens [4], [5]. Indeed, over the past 20 years, several new viral, bacterial, and eukaryotic/parasitic diseases have emerged in salmonid (*Salmo salar, S. trutta,* and *Oncorhynchus mykiss*) farming [2]. In particular, the occurrence of columnaris disease (caused by the opportunistic pathogen, *Flavobacterium columnare,* Bacteroidetes) has increased dramatically [3].

Traditionally, the evolution of pathogens is viewed as a reciprocal arms race competition between a pathogen and its host. Theories of virulence evolution predict that within-host growth of a pathogen, which is associated with virulence, is restricted by the availability of hosts [4]. This is referred to as the transmission-virulence trade-off. While obligate pathogens are dependent on their hosts and suffer from the transmission-virulence trade-off, opportunistic pathogens are able to survive and reproduce in environment, outside their hosts. In the latter case, the outside-host environment provides fluctuating selection pressures for opportunistic pathogens (e.g., predation, parasitism, and ecological changes), which may have correlated effects on pathogen virulence. Antagonistic species interactions between bacteria and their parasitic viruses (i.e. phage) are profoundly important regulators of bacterial abundance and traits [6]. Phage are the most abundant entities in the biosphere. They exceed the number of their host by ten-fold and thus have the capacity to control bacterial populations [7]. They also have a major impact on ecosystems and carbon cycling, especially in aqueous environments [8]–[10]. In the continuous arms race between phage and their hosts, bacteria need to evolve rapidly to avoid extinction [11]. Indeed, lytic phage have been shown to drive evolution in bacterial communities and to lead to strain diversification [12]–[15]. However, overlapping host-parasite interactions (e.g., bacterium-host and phage-bacterium) remain poorly understood. It is evident that theories regarding host-parasite interactions need to be expanded to involve three or more players in order to more accurately represent the evolution of virulence that is observed in complex environmental settings.

The ecological and evolutionary pressures that limit or increase the virulence of opportunistic bacterial pathogens are poorly known. In some cases, bacteria that gain resistance against lytic phage have a lower virulence, as has been demonstrated for *Serratia marcescens* [16], *Salmonella enterica* [17], and *Staphylococcus aureus* [18]. On the other hand, phage can invade the host as a prophage, and then modify and induce virulence of opportunistic bacteria by encoding virulence factors like the cholera toxin in *Vibrio cholera* [19]. This increases the pathogenicity of the host bacterium, and makes it a better competitor in the bacterial population [20], [21]. However, there is still very little information available regarding phage-host interactions that affect bacterial virulence under natural or intensive farming conditions. Moreover, the key question is, how do phage drive the evolution of opportunistic bacteria in the environment outside of a host. This aspect is especially important for understanding the emergence of opportunists, including opportunist saprotrophic pathogens which are able to replicate in, and transmit from, dead hosts. In these cases, the bacterial virulence is not necessarily limited by the virulence-transmission tradeoff.

*F. columnare* inhabits environmental microbial communities, but is also an opportunistic fish pathogen [22]. Disease outbreaks of columnaris disease rarely occur in nature, and the strains isolated from nature are less virulent than those isolated from fish farms [22]. Since the 1990s, columnaris infections have become increasingly more frequent and problematic in freshwater aquaculture, resulting in severe infections, increased mortality, and economic losses for fish farms producing salmonid fingerlings and fry [3]. This is mainly caused by the ability of *F. columnare* to survive in water, and outside of fish hosts for up to several months, thereby eluding antibiotic treatments [23]. *F. columnare* is also able to exploit dead fish material present in fish ponds for saprotrophic growth and as a means of transmission [23].

In nature and at fish farms, *F. columnare* encounters commensals and enemies from different trophic levels (e.g., protozoa, bacteria, viruses). However, studies of the interactions between *F. columnare* and other aquatic organisms present in the water body are scarce [24], [25]. Moreover, the impact of these interactions on bacterial virulence, and the mechanisms mediating bacterial virulence, are largely unknown. Under laboratory conditions, *F. columnare* exhibits three different colony morphologies, rhizoid (Rz), rough (R), and soft (S). The rhizoid morphotype is isolated in primary cultures from diseased fish, tank water, and biofilms. However, when the bacterium is cultured in the laboratory, or maintained under starvation, it loses its rhizoid morphology and manifests a parallel change in colony morphotype and decline in virulence and in susceptibility to phage infection [22], [26], [27], (Laanto et al., unpubl).

We have recently isolated and characterized phage capable of infecting the fish pathogen, *F. columnare* [28]. The results of previous studies, as described above, have demonstrated that lytic phage can act as a selective pressure against bacterial virulence, or can invade bacterium as a prophage and promote virulence. Based on the phage sensitivity of *F. columnare* [28], and the correlation between colony morphotype and virulence [26], [27], it is hypothesized that exposure of *F. columnare* to phage will cause a decline in bacterial virulence as a trade-off for acquiring phage resistance. Characterization of this phage-host relationship will provide novel insight into the virulence mechanisms of *F. columnare* in a fish host, as well as understanding on the infection dynamics of opportunistic pathogens outside their hosts. Moreover, studies of the complex host-parasite interactions that occur in intensive farming has the potential to facilitate our understanding of emerging new diseases, and to improve disease management in aquaculture.

## Materials and Methods

### Bacteria and Phage

Four previously isolated *F. columnare* strains from three different fish farms located in Central and Northern Finland were used in this study (Table 1) [28]. The bacteria were originally isolated from diseased fish and tank water obtained from fish farms as part of a disease surveillance project. To obtain isolates from fish, B67 and Os06, fish were euthanized by cutting the spinal cord prior to sampling. Isolates B185 and B245 were isolated from tank water. Standard culture methods were used to isolate bacteria, and included Shieh medium [29] for B185 and B245, Shieh medium supplemented with tobramycin [30] for B67, and AO-agar [31] for Os06. Pure cultures were stored at −80°C with 10% glycerol and 10% fetal calf serum. For the analyses performed, bacterial strains were grown in Shieh medium at room temperature (RT, approximately 24°C) with constant shaking (110 rpm). The genetic groups and phage susceptibility of B67, B185, and B245 were previously determined [28], while isolate Os06 was characterized in the present study (Table 1). Genetic grouping was based on the ribosomal intergenic spacer copy and length profile, and was performed as previously described [32]. In this study, the ancestral rhizoid morphotype is abbreviated, Rz, and the phage-induced morphotype rough is abbreviated, R.

**Table 1:** The *Flavobacterium columnare* strains and phage used in this study.

Bacterial strain*	Genetic group	Location	Source	Year Isolated	Phage used
B67	A	Farm L	Fish (*Salmo trutta*)	2007	FCL-1
B245	C	Farm V	Tank water	2009	FCV-1
B185	G	Farm L	Tank water	2008	FCL-2
Os06	G	Farm O	Fish (*Salmo salar*)	2006	FCL-2

* Bacteria and phage were previously characterized (28), except for Os06 which was genetically characterized in this study.

Three lytic phage used in the present study were previously isolated from tank water from two different fish farms in Central Finland (Table 1) [28]. These *F. columnare* phage are genotype-specific [28], and therefore, phage FCL-2 were used for two bacterial strains in the genetic group G (Table 1). Phage used were enriched with bacteria using the standard double layer method. Briefly, 300 µl fresh bacterial culture was mixed with 3 ml soft agar (0.7%) and 100 µl phage suspension and poured on Shieh agar plates. After 48 h incubation at RT, phage were isolated by adding 5 ml Shieh-medium on top of the agar plates that exhibited confluent lysis. The plates were shaken at 95 rpm for 24 h at 8°C, after which the phage lysates were filtered through a 0.45 µm Supor® Membrane (PALL Corporation) and stored at 4°C.

### Selection for Bacterial Colony Morphotypes with Phage

Phage that were used to select for phage-resistant morphotypes are listed in the Table 1 along with the corresponding bacterial strain. Phage lysates (50 µl of approximately 10^9^ to 10^11^ plaque forming units (PFU) ml^−1^) were spread on one half of a Shieh agar plate. Fresh bacterial culture (diluted to 10^−1^ and 10^−2^) was then spotted on both halves of the plate (in the presence and absence of phage). After 48 h, rough (R) *F. columnare* colonies that grew in the presence of phage were selected, cultured to assure the loss of the parental Rz growth, and then stored at −80°C for further analysis.

The number of phage needed to achieve growth of only rough colonies of *F. columnare*, was analysed by taking 100 colony forming units (CFU) of bacteria (400 CFU in the case of B185) and plating the bacteria on Shieh agar with and without phage (i.e. 100 µl phage suspension with known PFU ml^−1^ at three different dilutions). The phage suspension was first spread on a plate, dried, and then inoculated with a fresh culture of *F. columnare*. Bacterial CFU was estimated from the optical density of the liquid culture.

### Phage Resistance and the Presence of Phage in Rough (R) Morphotypes

Phage resistance of the rough (R) colonies that grew in the presence of phage was tested using a standard double layer method with slight modifications. Briefly, 300 µl fresh R morphotype bacterial culture was mixed with 3 ml soft agar and poured on Shieh agar plates. Five microliter aliquots from each of three dilutions of phage lysate were then spotted on the surface of the top agar. After 48 h at RT, the presence of plaques was recorded.

To determine if a phenotype change from ancestral rhizoid (Rz) to R was caused by lysogenic conversion (e.g., incorporation of phage into bacterium), the R morphotypes were tested for the release of phage into the culture medium. The lysogenic release of phage from R morphotypes into the supernatant was expected produce visible plaques on the Rz lawn. Three hundred µl of overnight grown Rz bacteria were plated with 3 ml soft agar (0.7%) on Shieh agar plates. R bacteria were centrifuged for 3 min at 13 000×g, diluted 10- and 100-fold, then 10 µl aliquots were spotted on the surface of the top agar. After 48 h at RT, the presence of plaques was recorded.

### Stability of Colony Morphotypes in Serial Culture

To evaluate the stability of the colony morphotype of Rz and R bacteria, ten serial culture transfers were performed for all strains and their morphotypes. For these experiments, cultures and samplings were not replicated, since the initial bacterial strains were considered to be replicates of each other. Briefly, bacteria were cultured in Shieh broth for 22–24 h, after which 1 ml of the grown bacteria was transferred into 5 ml of fresh Shieh medium. Ten serial transfers were performed for each bacterium. From each transfer, a sample was plated onto agar plates, and the proportion of colony morphotypes present were estimated. The frequencies of the colony types observed were further analyzed using factorial analysis of variance (ANOVA), with the day of measurement, strain identity, colony class, and day*strain and strain*colony class interactions evaluated as factors. In addition, the phage resistance of a single colony of Os06 that changed colony morphotype from R to Rz was tested as described above.

### Gliding Motility in Different Nutrient Conditions

Rz and R morphotypes of all bacterial strains were cultured on 2× concentrated Shieh, 1× (normal) Shieh, and 0.5× diluted Shieh agar plates. After 48 h, the colony diameter of 10 individual colonies were measured. Because the data did not fulfill the assumptions of normality, data were analysed using ANOVA on ranked data values. The sum of squares (SS) and mean squares (MS) were used to calculate test value H which was tested against chi square distribution with dfs corresponding to dfs of original treatment effects [33]. The analyses were performed using SPSS 20 (IBM).

### Virulence Experiments

Fish experiments were conducted according to the Finnish Act on the Use of Animals for Experimental Purposes, under permission ESAVI-2010-05569/Ym-23 granted for L-RS by the National Animal Experiment Board at the Regional State Administrative Agency for Southern Finland.

Unsexed, adult, disease-free zebra fish (*Danio rerio*) were obtained from Core Facilities (COFA) and research services of Tampere (Tampere University, Finland). These zebra fish were infected with both colony morphotypes of the four *F. columnare* strains by bathing. The infection method and bacterial dose used were developed and optimized in preliminary experiments (Kinnula et al. unpublished). The fish were individually challenged in 100 ml water with 1×10^7^ CFU ml^−1^ freshly grown bacteria for 30 min at 25–26.5°C. Each infection included eight replicates and four replicates of a negative control (i.e. fish exposed to sterile Shieh medium). After each challenge, fish were transferred into separate 3 liter aquaria, with one fish per aquarium with 1 liter bore hole water. Fish were monitored for 3 d for disease symptoms and mortality. Mortality data were analyzed using Cox regression conducted by SPSS 20 (IBM).

## Results

### Effect of Phage on Bacterial Colony Morphotype

**Figure 1:**
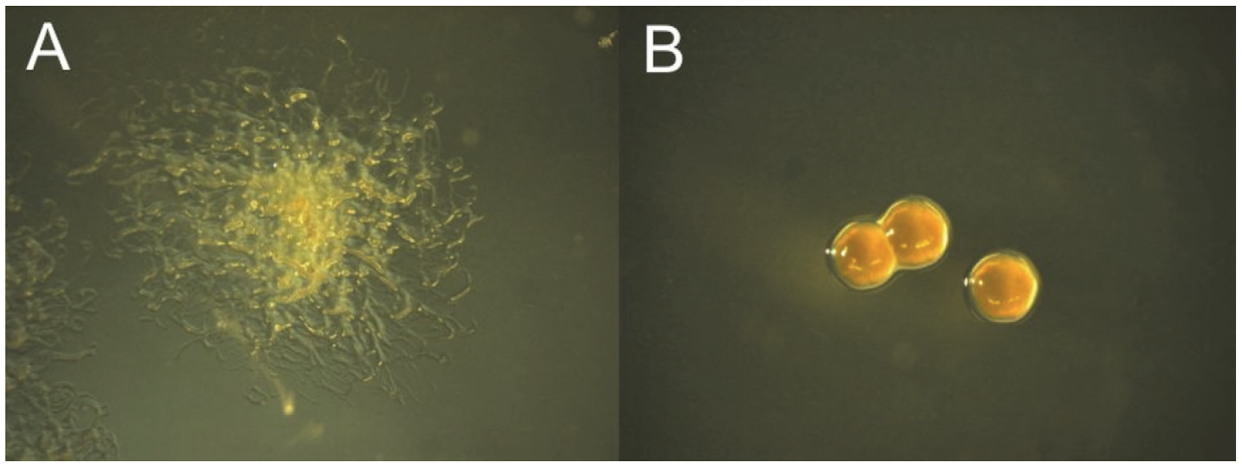
*Flavobacterium columnare* colony morphotypes. A) Ancestral rhizoid (Rz) colony and B) phage-resistant rough (R) colony on Shieh agar.

When cultured in the presence of phage, all bacterial strains studied here lost their rhizoid (Rz) colony morphotype. The phage resistant bacteria growing with the phage lysate had a rough (R) colony morphotype, with solid edges and small size (Figure 1). In contrast, the spontaneously formed rough morphotypes reported in previous papers [26], [27] have irregular edges with sporadic rhizoid protrusions. Moreover, these spontaneously formed rough morphotypes also exhibit gliding motility when cultured in low nutrient media (our unpublished data). For all bacterial strains evaluated, the R colonies were confirmed to be phage-resistant, and showed no signs of lysogenic release of phage into culture supernatant.

For each bacterium-phage combination assayed, approximately 100 (B67, B245, Os06) or 400 (B185) Rz colonies were observed to convert to phage-resistant R colonies over two days of culturing on agar plates at 22°C when the number of phages on each plate was 10^6^ or more. To change all the 100 ancestral Rz colonies to R colonies, 7.4×10^6^ PFU ml^−1^ phage FCL-1 and 6.5×10^6^ PFU ml^−1^ phage FCV-1 were needed for B67 and B245, respectively. Phage FCL-2 was not as efficient in causing a morphology change, with 2×10^9^ PFU ml^−1^ needed for Os06, and 3×10^9^ PFU ml^−1^ was needed for B185, to obtain phage-resistant colonies. For bacterial strains, B67, B245, and Os06, colony counts on plates with and without phage were similar. However, for B185, approximately 50% fewer total colonies were observed to grow in the presence of phage compared with a parental phage-free control. This may be due to the high cost of developing phage resistance in this strain, possibly by mutation.

### Gliding Motility in Different Nutrient Conditions

**Figure 2:**
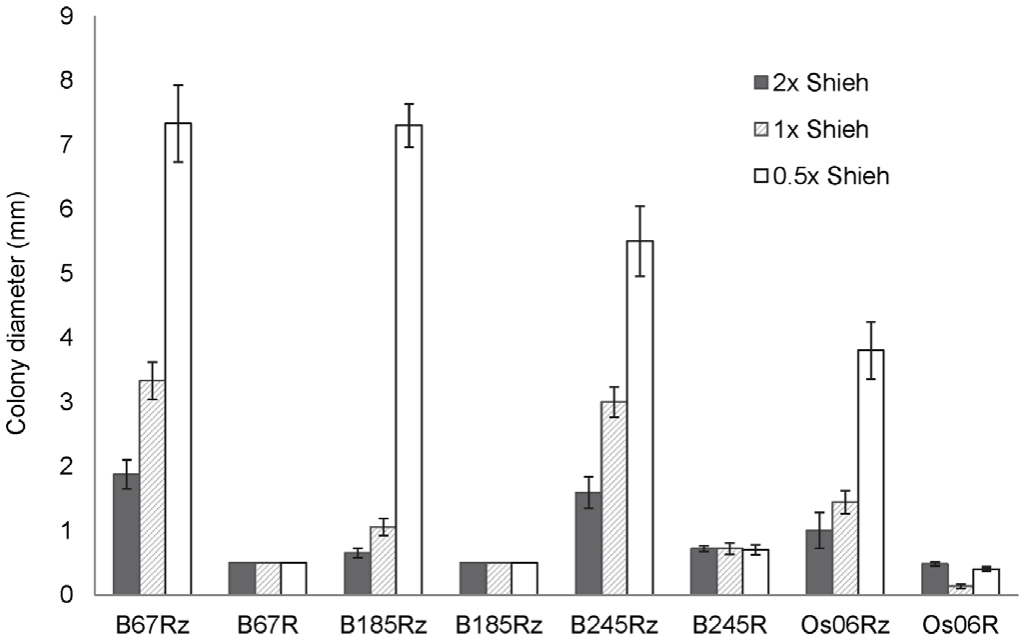
Gliding motility of colony morphotypes with different nutrient concentrations. Colony diameter (mm) was used to indicate the capacity for gliding motility of the *Flavobacterium columnare* rhizoid (Rz) and rough (R) colony morphotypes under different nutrient conditions. Motility is presented as the mean colony diameter (mm±SE) obtained from ten colonies grown for 48 h on 2× concentrated, normal (1×), and diluted (0.5×) Shieh agar. Colony diameter was found to be significantly affected by colony morphology (H = 129.6, p<0.001), and by nutrient concentration (H = 10.6, p<0.001, colony*nutrient interaction H = 20.1, p<0.001).

The colony diameter of Rz and R colonies grown on agar plates with different nutrient concentrations were measured to characterize the ability of the morphotypes to move by gliding. Overall, colony diameter was found to be significantly affected by colony morphology (H = 129.6, p<0.001) and nutrient concentration (H = 10.6, p<0.001), and in addition to main effects, also significant interaction between colony morphology and nutrient concentration was found (H = 20.1, p<0.001) (Figure 2).

### Stability of Colony Morphotypes in Serial Culture

**Figure 3:**
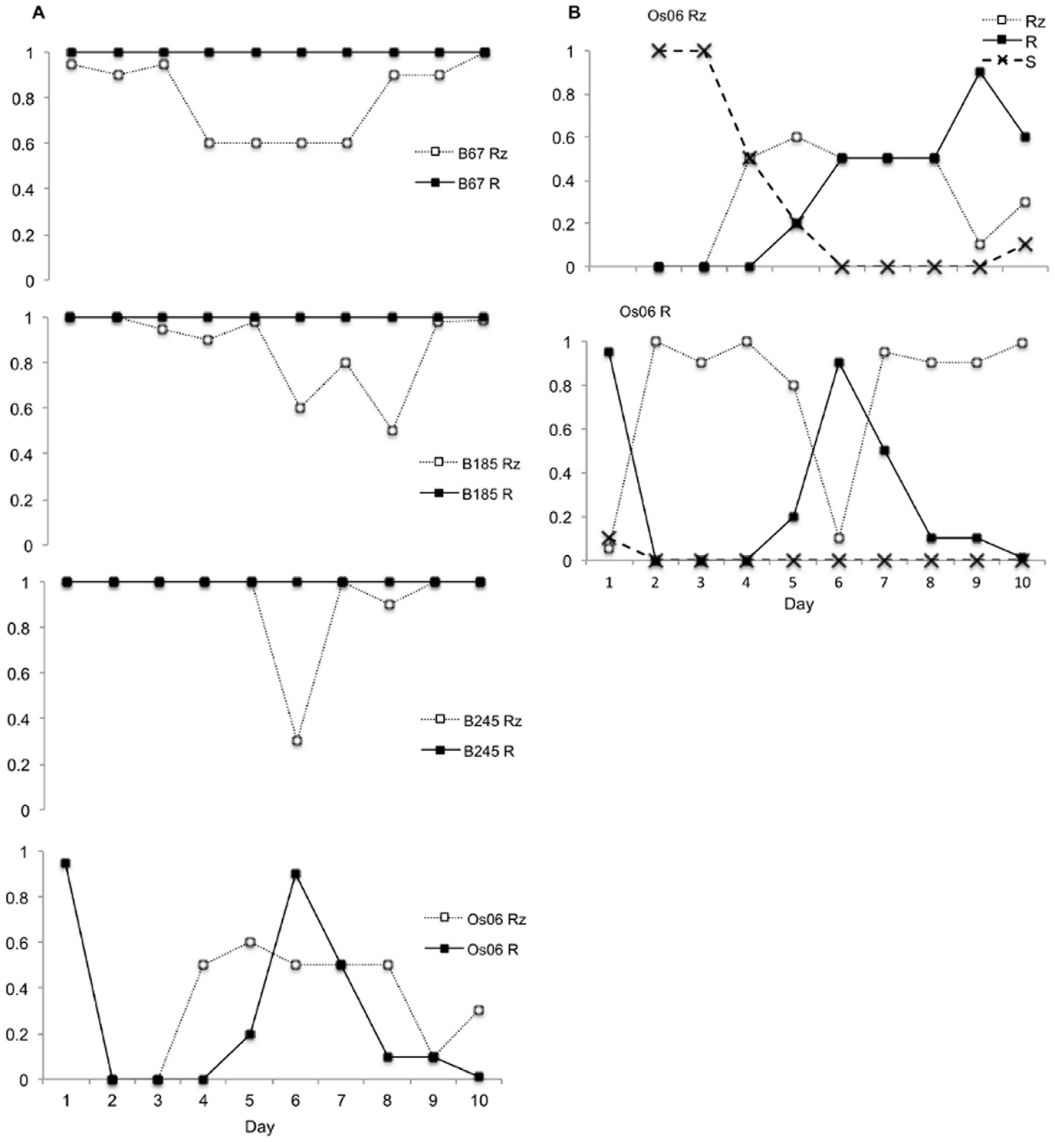
Proportion of *Flavobacterium columnare* colony morphotypes in serial culture. The proportion of colony morphologies (Rz = rhizoid, R = rough, and S = soft) in serial cultures of ancestral rhizoid and phage-resistant rough morphotypes of four *F. columnare* strains were estimated from pure cultures taken from each passage in Shieh broth. ND = not determined, but the proportion of Rz colonies was >50%. A) The proportion of ancestral Rz and phage-resistant R morphotypes that maintained their morphotype in serial culture, B) The fluctuation in the proportion of different morphotypes detected in Rz and R cultures of strain Os06.

The stability of the two colony types during serial culture was found to depend on the strain identity and the strain colony morphotype (strain identity: Wald χ2 = 364.5, df = 3, p<0.001; strain×colony morphotype: Wald χ2 = 17.5, df = 4, p = 0.002) (Figure 3a). For example, the phage-resistant strains, B67, B185, and B245, almost completely maintained their R colony morphology during the ten serial transfers. However, among the ancestral Rz morphotype spontaneous R colonies did appear. These spontaneously formed R colonies had irregular edges with some rhizoid protrusions, thereby differentiating them from the phage-resistant R colonies. The ancestral Rz and phage-resistant R morphotypes of strain Os06 were the most unstable, as they changed morphotypes in each culture. The soft (S) morphotype in particular was observed frequently (Figure 3b). Furthermore, the Os06 R morphotype did change back to the Rz morphotype and maintained its phage resistance.

### Virulence Experiments

Virulence of the ancestral Rz morphotype was compared to the phage-resistant R type using a zebrafish infection model. The Rz morphotype was associated with a significantly higher mortality rate than the phage-resistant R morphotype (Cox regression, Wald = 27.4, df = 1, p<0.001, Table 2). Moreover, in most cases, the R morphotype bacteria were completely non-virulent, but experiments involving B185 R did result in the death of one fish. The virulence of the bacterial strains also significantly differed from each other (Cox regression, Wald = 13.1, df = 1, p<0.001). For example, the Rz morphotype of B185 and B67 were the most virulent, and resulted in the death of all eight fish tested within 24 h. In contrast, experiments with B245 resulted in the death of 4/8 fish, and for Os06 2/8 fish died (Table 2). The fish that died showed no obvious external signs of columnaris disease, indicating that the rapid progression of the disease can cause a symptomless death, possibly by blocking the gill surfaces. The remaining fish did not develop any disease symptoms during the three day post-infection monitoring.

**Table 2:** Zebrafish mortality caused by *Flavobacterium columnare* strains with ancestral rhizoid (Rz) versus phage-induced rough (R) morphotypes.

	Fish mortality^a^ caused by morphotypes	
Strain	Ancestral Rz	Phage-resistant R
B67	100%	0%
B185	100%	12.5%
B245	50%	0%
Os06	25%	0%

a Zebrafish (n = 8) were individually tested for each morphotype.

## Discussion

Antagonistic co-evolution between a bacterial host and its parasitic phage can represent a selective pressure for bacterial virulence. In this study, the ability of specialized parasitic phage to modulate the virulence of opportunistic pathogen outside its vertebrate host was investigated by exposing four strains of the opportunistic fish pathogen, *Flavobacterium columnare*, to corresponding lytic phage. When bacteria manifesting an ancestral rhizoid (Rz) morphotype were cultured in the presence of lytic phage, the colony morphotype changed to rough (R), and was accompanied by a loss of virulence and gliding motility. These results demonstrate that phage can cause phenotypic changes in *F. columnare* outside of the fish host, and thus, modulate its virulence.

While there are various mechanisms that can select for high virulence of pathogens, including within-host strain competition [34] and formation of persistent spores [35], opportunistic pathogens diverge fundamentally from the assumptions of epidemiological models. Opportunistic pathogenic bacteria are exposed to different environments during their life cycle, and they can have different strategies for living inside and outside a host [36]. Outside of a host, bacteria can be exposed to predators and parasitic phage, and they also need to compete for resources. On the other hand, within host growth requires traits for overcoming the immune defense of a multicellular host while growing otherwise in an enemy-free, high-resource host tissue. These conditions can either decrease or increase infectivity. Furthermore, opportunistic saprotrophic bacteria can reproduce and transmit from dead hosts, and can actively grow outside of a host as part of the normal aquatic food web. Indeed, *F. columnare* transmits most efficiently from dead hosts (making high virulence traits beneficial), and can survive for long periods (at least five months) in water outside of a host [22], [23]. These traits may explain why columnaris disease has become problematic in fish farms [3]. However, the impact of the selective pressures generated by the environment outside of a host on the virulence of *F. columnare* has not yet been elucidated.

Based on the dramatic loss of virulence associated with phage-resistant *F. columnare* exhibiting a rough colony morphotype, we hypothesize that virulence is related to gliding motility in this bacterium. Accordingly, motility and virulence have been found to be connected in many bacteria [37], [38]. However, unlike many other bacteria, *F. columnare* does not have flagella or pili. Rather, it moves on surfaces by gliding, a motility characteristic associated with flavobacteria. Moreover, the flavobacterial gliding motility system has been shown to be orthologous to the Por secretion system (PorSS) of virulence factors in *Porphyromonas gingivalis* [39]. The corresponding genes needed for gliding/PorSS also exist in *F. columnare* [40], thereby suggesting that the motility apparatus may play a role in the secretion of virulence factors also in this species. This hypothesis would also be consistent with the motility-dependent virulence of this bacterium [26], [27]. Phage may reduce bacterial motility by using the bacterial motility apparatus as a receptor [41]–[43]. Therefore, to resist phage infection, bacteria may mutate, or down-regulate, the expression of the motility apparatus to impair motility (and virulence as well if it is coupled with motility). In other bacterial species, the negative effect of phage resistance on bacterial virulence outside a host has been observed [16]–[18]. In contrast, traditionally in the phage-host literature, more emphasis has been given to the capacity for phage to provide new genetic material and virulence factors for a host bacterium, thus increasing the fitness of the host bacterium [44], [21]. Phage resistance may, however, have trade-offs with respect to bacterial growth [18], and this may be true for *F. columnare* as well (Zhang et al., unpublished). Therefore, it is essential to recognize that phage can have both negative and positive effects on the virulence traits of their hosts, with the former highlighted in this study.

In addition to changes in colony morphotype selected by the presence of phage, spontaneous morphology changes from Rz to R in serial cultures of *F. columnare* were also detected, a phenomenon that has been previously reported [26], [27], [45], [46]. However, the appearance and biological significance of spontaneous R morphotypes are different than those of phage-resistant R types. For example, spontaneously formed R colonies appear during serial culture in the laboratory, they often exhibit weakly rhizoid or irregular edges, and they retain their gliding motility in low nutrient conditions. In contrast, phage-resistant R colonies are small with solid edges. The number of Rz morphotype colonies has a tendency to decrease in the bacterial population during culture (see Figure 3), indicating that colony morphotype is probably regulated by gene expression and phenotypic changes in response to environmental conditions. However, in the three virulent bacterial strains studied (e.g., B67, B185, and B245), the Rz morphotype was maintained at higher numbers during serial culture, suggesting that selection for the virulent Rz phenotype in these strains is strong, even under laboratory conditions. Also, the phage resistant R morphotypes of the same strains were stable, indicating that phage resistance may involve genetic effects (e.g., mutation of the phage receptor), which are not rapidly reversed under laboratory conditions, especially if phage cause a strong selective pressure as in case of B185 and FCV-2. Altogether, the general instability of the bacterial colony morphotype seems to be limited to certain strains (e.g., Os06), possibly to allow these bacteria to rapidly switch between phenotypes as necessary [47], [48]. This would increase the chances of survival in an unpredictable environment, rather than having changes in phenotype/expression induced by environmental cues. Moreover, rapid shuffling between morphotypes in a low-virulence strain may be less costly than shuffling in more virulent strains, as less virulent bacteria (e.g., Os06) may not suffer from a loss of motility since they may not always be dependent on fish hosts for their survival. Furthermore, it can be assumed that less virulent strains living outside of a host are more frequently exposed to trophic interactions, and therefore the ability to change morphotype rapidly is beneficial for avoiding enemies and adapting to changing environmental conditions [22]. The history of *F. columnare* as an environmental bacterium is consistent with these hypotheses.

The observation that phage resistance correlates with loss of virulence is especially important when designing novel treatment methods for intensive farming. Columnaris disease is an economically significant fish disease in freshwater aquaculture around the world. In addition, columnaris disease is an epidermal disease and transmits via water. Therefore, based on previous studies that have shown phage therapy to be successful for other fish diseases [49]–[51], and the potential for phage to be applied directly to tank water, we hypothesize that phage represent a valuable disease targeting strategy. Moreover, if phage use the gliding motility apparatus of *F. columnare* as a receptor, as suggested by the results of the present study, phage-host interactions would be mediated at the surface of the skin and gills of infected fish (or in the biofilms of the fish tank). Therefore, as fingerling fish are reared in relatively small water volumes, the amount of phage needed for treatments would be at a manageable level. In addition, the development of phage resistance would not hamper the success of the therapy, if the bacteria maintain their R morphotype as efficiently in the fish farming environments as they did in the laboratory. Additional studies will be needed to confirm these hypotheses, and to further elucidate the details of phage-host interactions.

In this study, zebrafish was used as the model organism rather than rainbow trout or other salmonids, which are common hosts of columnaris disease at fish farms. Therefore, it can be argued that the use of a model organism that is not the natural host of the studied disease could produce different outcomes [52]. However, *F. columnare* has a wide host range, and correspondingly, has been isolated from European graylings (*Thymallus thymallus*), whitefish (*Coregonus lavaretus*), bream (*Ambramis brama*), pikeperch (*Zander lucioperca*), and perch (*Perca fluviatilis*) in Fennoscandia [22], [32]. Furthermore, the use of zebrafish for *F. columnare* infections has previously been described [53], and our unpublished studies suggest that zebrafish can be used as a reliable model for studying columnaris disease instead of rainbow trout (Kinnula et al., unpublished). There are other advantages to using zebrafish as well. Zebrafish is established as model organisms for a wide range of experimental studies, disease-free fish are available year round, and they tolerate laboratory conditions better than rainbow trout. However, it will be important to confirm the phage-host interactions identified in the present study in salmonids, especially for the development of therapeutic applications.

To conclude, parasitic phage of *F. columnare* can select against bacterial virulence and motility, as indicated by colony morphology, in an environment outside a fish host. By studying phage-driven selection pressures and their impact on bacterial virulence, we have the opportunity to gain insight into the virulence factors of *F. columnare* and understand how its virulence evolves on both ecological and molecular scales. To our knowledge, this study represents the first report of the effects of phage-host interactions on a commercially important fish pathogen where phage resistance directly correlates with a decline in bacterial virulence. The phage-driven loss of virulence observed also supports the hypothesis that antagonistic co-evolution can reduce the virulence of opportunistic pathogens outside of a host due to the associated costs of defending against parasitic or predatory enemies [16]. Our results also indicate that further characterization of phage-host interactions is needed, particularly in regard to impact of phage on the evolution of virulence in opportunistic pathogens that affect intensive farming, and to develop applications for disease control.
